# Drugs Metabolism-Related Genes Variants Impact on Anthracycline-Based Chemotherapy Induced Subclinical Cardiotoxicity in Breast Cancer Patients

**DOI:** 10.3390/ijms26094051

**Published:** 2025-04-25

**Authors:** Domas Vaitiekus, Gintare Muckiene, Dovydas Verikas, Audrone Vaitiekiene, Skaiste Astasauskaite, Rolandas Gerbutavicius, Agne Bartnykaite, Rasa Ugenskienė, Renaldas Jurkevičius, Elona Juozaitytė

**Affiliations:** 1 Clinic of Oncology and Hematology, Institute of Oncology, Faculty of Medicine, Lithuanian University of Health Sciences, LT-44307 Kaunas, Lithuania; 2 Clinical Department of Cardiology, Medical Academy, Lithuanian University of Health Sciences, LT-44307 Kaunas, Lithuania; 3 Laboratory for Automation of Cardiovascular Investigation, Institute of Cardiology, Lithuanian University of Health Sciences, LT-44307 Kaunas, Lithuania

**Keywords:** anthracycline cardiotoxicity, chemotherapy heart damage, doxorubicin cardiomyopathy, subclinical heart damage, *SULT2B1* rs10426377, *UGT1A6* rs17863783, *CBR1* rs9024, *CBR3* rs1056892, *NCF4* rs1883112, *CYBA* rs1049255

## Abstract

Breast cancer is the most common cancer in women worldwide. Anthracyclines (doxorubicin, epirubicin, daunorubicin, idarubicin) are among the most used drugs for the treatment of breast cancer. Unfortunately, anthracyclines cause cardiotoxicity, which is a limiting factor for its use, and the lifetime cumulative dose of anthracyclines is the major risk factor for cardiotoxicity. In our study, we focused on acute and subacute heart damage. One of the main factors is a genetic predisposition, which determines individual susceptibility to anthracycline cardiotoxicity. The main idea of this study was, for the first time, to evaluate drug metabolism-related genes as a risk factor for developing cardiovascular toxicity in breast cancer patients. The main objective of our study was to identify the impact of drug metabolism-related gene SNPs on the development of subclinical heart damage during and/or after doxorubicin-based chemotherapy in breast cancer patients. The data of 81 women with breast cancer treated with doxorubicin-based chemotherapy in an outpatient clinic were analyzed, and SNP RT-PCR tests were performed. The drug metabolism-related gene variants *SULT2B1* rs10426377, *UGT1A6* rs17863783, *CBR1* rs9024, *CBR3* rs1056892, *NCF4* rs1883112, and *CYBA* rs1049255 did not reach a statistically important impact on ABCC in multivariate logistic regression analysis. However, we identified that *NCF4* rs1883112 had a risk reduction tendency for ABCC (OR = 0.49, 95% CI 0.27–0.87, *p* = 0.015). Our findings suggest that some SNPs, such as *NCF4 rs1883112*, may be associated with a reduced risk of cardiotoxicity, while no variants in this study showed a statistically significant increased risk. Even though, *NCF4* rs1883112 showed a risk reduction tendency, suggesting the potential for personalized risk stratification. We can conclude that multiple genes are involved in ABCC, with different impacts, and it is unlikely that there is a single driver gene in ABCC pathogenesis.

## 1. Introduction

Breast cancer is the most common cancer in women worldwide. In 2017, Ellison et al. found that breast cancer’s 5-year survival in Canada exceeded 80% [1]. Anthracyclines (doxorubicin, epirubicin, daunorubicin, idarubicin) are among the most commonly used drugs for the treatment of leukemia, lymphoma, and various solid tumors in adults and children, including breast, ovarian, lung, and sarcoma. They are on the World Health Organization’s list of essential medicines. Anthracyclines, especially doxorubicin, are the cornerstone of breast cancer treatment. The exact mechanism of anthracycline-induced cardiotoxicity remains unclear. One of the first hypotheses was that anthracyclines enhance redox reactions. DNA is vulnerable to the production of reactive oxygen species, the levels of which can also be increased by free intracellular iron [2]. Doxorubicin and iron complexes form a toxic radical, which enhances the effects of oxidative stress and develops mitochondrial dysfunction [3]. Doxorubicin inhibits topoisomerase II alpha, causing chemical and oxidative DNA damage. This further disrupts DNA and RNA synthesis and induces apoptosis in cells. Topoisomerase II is now thought to be one of the major mediators of cardiotoxicity. Both mechanisms of anthracycline action increase free radicals in the endothelium due to oxidative stress. The myocardium is sensitive to free radicals due to low levels of antioxidant enzymes in the heart. Exposure to reactive oxygen species causes peroxidation of cardiomyocyte membrane lipids [4]. The use of anthracyclines is limited by dose-dependent adverse effects. In many studies, a total dose of 400 mg/m^2^ has been shown to increase the risk of heart failure to 5%, with 550 mg/m^2^ to 26% and 700 mg/m^2^ to 48%, respectively [5,6].

Compared with other causes of heart failure, the prognosis for anthracycline-related cardiomyopathy is deterioration, so early diagnosis is relevant. Cardinale et al. performed a study that followed 2625 anthracycline-treated patients for 5.2 years. The results showed a cardiotoxicity of 9%. Cardiotoxicity was proven in 98% of patients who developed cardiotoxicity within the first 12 months of medication. Cardiotoxicity was defined in this study as a reduction in left ventricle ejection fraction (LVEF) of >10% from baseline and less than 50% overall [7,8]. There are three types of anthracycline-related cardiotoxicity: acute, subacute, and late/delayed [8]. Acute heart failure develops in <1% of patients in the first week after chemotherapy. It is characterized by supraventricular tachycardia, transient LV dysfunction, and electrocardiographic changes. Acute heart damage is usually reversible and dose-independent. Subacute effects occur within the first year after treatment. Symptoms of heart failure and LV dysfunction are the most observed. Late effects manifest themselves after years of treatment and are dose-dependent and cause dilated cardiomyopathy. Many affected patients are asymptomatic; however, they remain at risk of developing heart failure years later [6]. Cardiotoxicity-related symptoms may include dyspnea, chest pain, palpitations, leg swelling, and dizziness. In our study, we focused on acute and subacute heart damage.

It is relevant to evaluate subclinical heart damage because we rarely see clinical signs of heart failure in acute and subacute types. Subclinical ejection fraction decrease is not widely discussed in the literature. According to the American Society of Clinical Oncology (ASCO) guidelines and other publications, there are risk factors for anthracycline-based chemotherapy cardiotoxicity (ABCC), including lifetime cumulative dose, infusion regimen, or any disease that raises cardiac susceptibility [5]. One of the main factors is genetic predisposition, which determines individual susceptibility to anthracycline cardiotoxicity. The main idea of this study was, for the first time, to evaluate drug metabolism-related genes as a risk factor for developing cardiovascular toxicity in breast cancer patients.

Anthracycline cardiotoxicity is greatly influenced by genes associated with drug metabolism, which are important determinants of individual susceptibility to anthracycline toxicity [9,10]. Anthracyclines are mainly metabolized by enzymes belonging to the cytochrome P450 (CYP) family, such as CYP2D6, CYP3A4, and CYP3A5 [11]. Variants in these genes can alter enzymatic activity, resulting in large individual variability in anthracycline metabolism and associated toxicity [12]. In addition to CYP enzymes, other genes, such as glutathione-S-transferase (GST) and multidrug resistance-associated protein (MRP), are also involved in anthracycline metabolism and cardiotoxicity [13]. The impact of these gene mutations or polymorphisms on anthracycline-induced cardiotoxicity has been the subject of intense investigation, with conflicting results reported in the literature [14,15].

One of the important enzymes studied in anthracycline-induced cardiotoxicity is sulfotransferase family cytosolic 2B member 1 (SULT2B1), which is involved in sulfation, a phase II metabolic process responsible for the sulfate conjugation of various drugs and endogenous compounds [9]. Another key phase II process is glucuronidation, carried out by enzymes such as UDP-glucuronosyltransferase 1A6 (UGT1A6), which facilitates the elimination of potentially toxic metabolites. Altered glucuronidation may lead to the accumulation of toxic anthracycline metabolites [11,12].

In addition, enzymes such as *carbonyl reductases* (CBRs), members of the short-chain dehydrogenase/reductase (SDR) family, catalyze the reduction of carbonyl-containing compounds including anthracyclines, potentially contributing to metabolite-related cardiotoxicity [16,17,18].

Another pathway of interest is the NADPH oxidase system, which contributes to oxidative stress. Variants in genes such as NCF4 (neutrophil cytosolic factor 4), CYBA (cytochrome b-245 alpha chain), and CYBB (cytochrome b-245 beta chain) encode components of this multicomponent enzyme complex, which generates reactive oxygen species by transferring electrons from NADPH to molecular oxygen [19,20]. The CYBA gene encodes *p22phox*, a critical subunit of NADPH oxidase, which is also essential for this activity. 

Therefore, it is important to identify specific gene variants associated with drug metabolism that are associated with an increased risk of cardiotoxicity in breast cancer patients receiving anthracycline-based chemotherapy. Such information can support individualized treatment strategies and improve treatment outcomes [9]. The main idea of this study was, for the first time, to evaluate drug metabolism-related genes as a risk factor for developing cardiovascular toxicity in breast cancer patients.

## 2. Results

Table 1 presents the cancer-related features of the participants. The age of the ABCC patients ranged from 34 to 76 years, with a mean of 52.870 years (SD = 10.248), while the control subjects were aged between 32 and 73 years, with a mean of 54.840 years (SD = 8.925). Statistically significant differences (*p* ≤ 0.0001) were identified between the ABCC patients and controls in terms of arterial hypertension and a family history of cardiovascular disease. Among the ABCC patients, 23 (76.7%) had arterial hypertension, and 16 (53.3%) reported a positive family history of cardiovascular disease. In comparison, hypertension was present in nine (17.6%) of the control group, and four (7.8%) controls had a family history of cardiovascular conditions. No statistically significant group differences were found in the prevalence of diabetes mellitus, dyslipidemia, smoking status, or BMI. Diabetes mellitus was diagnosed in 13 (16.0%) breast cancer cases (23.3% of ABCC patients and 11.8% of controls). Dyslipidemia was observed in 19 (23.5%) individuals across both groups. A total of 16 participants reported smoking, with a higher proportion among the ABCC patients (26.7%) compared to the controls (15.7%). In both groups, over half of the individuals had a BMI equal to or exceeding 25 kg/m^2^.

No significant variation was found between cases and controls regarding pathological stage (pTNM), side of breast affected, tumor grade, molecular subtypes, or hormone receptor expression (*p* > 0.05). The most common pathological stages were IIA and IA, based on the TNM classification. Most cancers occurred on the right breast (53.3% in ABCC patients vs. 60.8% in controls). Histological analysis revealed that 79% of tumors were grade 2 (G2); only one case of grade 1 (G1) cancer was recorded, found in a control subject. Luminal A emerged as the predominant tumor subtype, present in 29 (35.8%) of the breast cancer patients.

### Drug Metabolism-Related Genes Are Linked to ABCC

**Figure 1:**
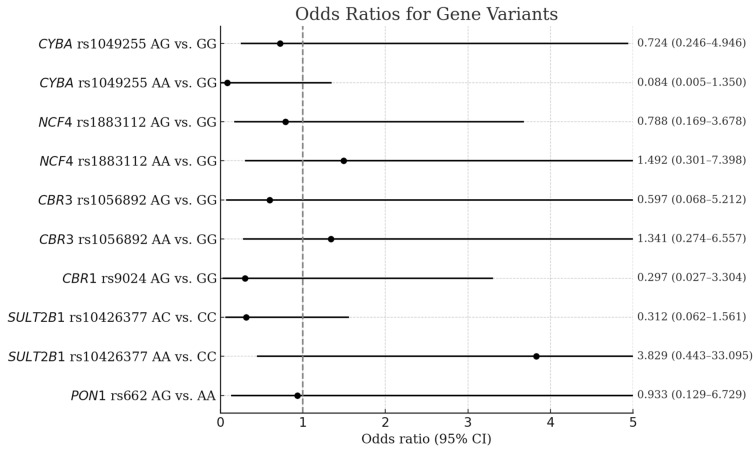
Forest plot of the association between SNPs and the risk for ABCC.

The frequencies of alleles and genotypes of drug metabolism-related gene SNPs in the controls and patients with ABCC and their association with the risk of ABCC are shown in Table 2. The genotype frequencies obtained for all SNPs studied agreed with the Hardy–Weinberg equilibrium; no difference between distribution was found (*p* > 0.05). The *NCF4* rs1883112 AG genotype was associated with a risk reduction in ABCC (OR 0.48, CI 0.25–0.93; *p* = 0.030). In the same way, when the dominant inheritance was considered, the genotypes AA and AG were less common in the ABCC group (OR 0.49, 95% CI 0.27–0.87; *p* = 0.015). Moreover, patients with the *CYBA* rs1049255 genotype AG were associated with a reduced number of ABCC among this study’s participants (OR 0.50, 95% CI 0.26–0.97; *p* = 0.041). Thus, considering dominant inheritance, the genotypes AA and AG were more recurring in the ABCC group (OR 0.57, 95% CI 0.35–0.94, *p* = 0.029). Other genotypes were distributed similarly in the control and ABCC groups. The majority of genes were found to have a tendency to reduce the risk of ABCC development; however, only *NCF4* rs1883112 and *CYBA* rs1049255 were shown to be significantly associated with a reduced risk of ABCC development, while the others did not have such an association. All SNPs analyzed together did not show significant associations with ABCC (Figure 1). Moreover, after adjusting for risk factors, all significant associations from univariate analysis resulted in the loss of significance (Table 3). In all tested metabolism-responsible gene SNPs, in the multivariate logistic regression analysis, we can observe a profound impact on ABCC when arterial hypertension and cardiovascular disease in the family history are present.

## 3. Discussion

In this prospective study, we analyzed the genetic features and clinical risk factors in early phase subclinical heart damage of 81 patients who were treated with anthracyclines for breast cancer. While no variants showed statistically significant associations after multivariate adjustment, *NCF4 rs1883112* demonstrated a tendency toward a protective effect. These findings suggest a potential role of oxidative stress pathway genes in modifying cardiotoxicity risk, warranting further investigation. Most gene polymorphisms were never evaluated in this population; therefore, our study presents better insights into metabolism-related genes and their correlation with cardiotoxicity. The results will lay the ground for further studies in genetic oncology.

### 3.1. PON1 rs662

As for the *PON1* gene, it is known that oxidative stress, which occurs in free superoxide radical aggregation, membrane lipid oxidation, and DNA damage, is associated with cancer pathogenesis, and PON1 is responsible for the elimination of various toxins and carcinogens [21,22,23]. Our results suggest that the GG genotype was not prevalent in our cohort; thus, a comparison with other studies cannot be performed. In 1997, the Odawa et al. study found an association of the *PON1* gene G allele with heart damage. They identified that Japanese G allele carriers with type 2 diabetes have a higher risk of developing coronary heart disease compared to carriers of allele A [24]. The *PON1* gene is associated with cardiovascular diseases, oxidative stress in the endothelium, and an increase in free radicals caused by anthracyclines. The myocardium is known to be sensitive to free radical exposure due to low levels of antioxidant enzymes in the heart. Peroxidation of cardiomyocyte membrane lipids occurs under the influence of reactive oxygen species [21]. In another study in 2013, Hassan et al. identified the GG genotype in a Saudi Arabia population, which is associated with coronary artery disease and is independent of identified risk factors, such as age, gender, smoking, obesity, and diabetes [25]. In 2013, a meta-analysis involving 28 studies was conducted by Liu et al. The G allele was shown to be associated with increased ischemic stroke risk in the general population [26]. In 2014, Geng reported that rs662 could be used as a prognostic factor in patients with metastatic cancer treated with epirubicin, oxaliplatin, and a combination of 5-fluorouracil. In the results obtained, the *PON1* gene rs662 polymorphism AA and AG genotypes were significantly associated with poor overall survival [27]. To the best of our knowledge, this is the first study to analyze the *PON1* gene rs662 polymorphism and its relationship with ABCC in the Caucasian population.

### 3.2. PON1 rs3735590

For the first time, our study analyzed the association of heart damage with the *PON1* rs3735590 polymorphism in patients with breast cancer treated with doxorubicin. No statistically significant results were obtained. Although there is a tendency in the results that this gene may be involved in the cardiotoxic effects of doxorubicin, further studies with a larger number of subjects are needed. In our study, the cohort had only the CC genotype; therefore, no analysis was performed. Talking about the *PON1* rs3735590 polymorphism, a total of five studies have been performed, in four of which the T allele has been associated with a protective effect against atherosclerosis [28], and the CT and TT genotypes were associated with a lower risk for aortic valve calcinosis and longer survival [29,30]. Paraoxonase 1 imbalance plays a relevant role in the pathogenesis of dyslipidemia. Dyslipidemia is a risk factor for aortic valve calcination and cardiotoxicity. Decreases in paraoxonase 1 activity have also been observed in patients with diabetes mellitus and myocardial infarction. A study by Zhang et al. found that the C allele is an independent risk factor for coronary heart disease [31].

### 3.3. SULT2B1 rs10426377

The SULT2B1 enzyme (sulfotransferase family cytosolic 2B member 1) is engaged in drug sulphate conjugation [7]. The development of hazardous anthracycline metabolites might be caused by altered glucuronidation [16,17]. Our results are among a female-only population; despite this, we can also see close to statistically significant results. Although *SULT2B1* rs10426377 did not approach statistical significance, it did show a trend toward risk reduction [11,32]. We observed, in the multivariate logistic regression analysis, that AA compared to CC carriers had OR = 0.084 for ABCC and *p* = 0.08; these results replicate the published data about possible risk reduction, with no statistical significance in the female population. Visscher et al. conducted a study with 218 patients from a separate cohort [11]. ABCC was characterized as early- or late-onset left ventricular dysfunction evaluated by echocardiography (shortening fraction, SF) or symptoms needing intervention, according to CTCAEv3 (Common Terminology Criteria for Adverse Events). ABCC has been identified and replicated in independent pediatric patient populations, where *SULT2B1* rs10426377 was close to being significantly associated with ABCC risk reduction; however, the *SULT2B1* variant rs10426377 had an effect in males but not in females (OR = 0.82 vs. 0.35) [11].

### 3.4. UGT1A6 rs17863783

UGT1A6 is a UDP glucuronosyltransferase with a potential role in anthracycline metabolism [12]. In our case, the population is too small to give any conclusion of this SNP in our study population. Because of the limited number of gene variations, we were unable to draw any conclusions. In a few independent pediatric patient population studies [9,11,12], UGT1A6 was found to have a connection with an elevated risk of ABCC. The drug detoxification glucuronidation pathway is where UGT1A6 plays a role. Reduced UGT1A6 glucuronidation of anthracycline metabolites might result in hazardous metabolite buildup in *UGT1A6* rs17863783 carriers, increasing the risk of ABCC. Another important study was conducted with hematopoietic stem cell transplant (HSCT)—patients who developed clinically relevant cardiomyopathy 1 year or later after HSCT. This SNP was not linked to late-onset cardiomyopathy risk. Early-onset cardiomyopathy was predicted with fair accuracy (AUC = 0.76, 95% CI 0.68–0.83) by a combination of demographic, therapeutic, and clinical factors, while late cardiomyopathy was poorly predicted (AUC = 0.59, 95% CI 0.53–0.67) [33].

### 3.5. CBR3 rs1056892

Carbonyl reductases (CBRs) CBR1 and CBR3 are members of an enzyme family that catalyzes numerous chemical molecules, including anthracycline drugs: doxorubicin or daunorubicin [18]. These proteins, by catalyzing the reduction in chemotherapy drugs to toxic alcohol composites, may strongly influence metabolic processes in the cells. For example, the deletion of *CBR1* reduces the production of alcohol metabolites and sensitivity to the cardiotoxic effects of chemotherapy [34]. We found that the AG genotype was associated with an increased risk of ABCC; however, it was not significant due to the relatively small sample. Blanco et al. studied 30 patients who developed congestive heart failure after being treated with anthracyclines and 115 control patients. *CBR3* rs1056892 was shown to have a stronger tendency than other variations to increase the risk of cardiac damage [35]. The *CBR3* rs1056892 G allele was noticed to make 2.6-fold more harmful doxorubicin per unit of time than the *CBR3* rs1056892 A allele. These results showed a strong tendency of CBR3 rs1056892, more than other variants, potentially increasing the risk of heart injury, resulting in CHF (OR = 8.16, *p* = 0.056 for GG vs. AA; OR = 5.44) [35]. The *CBR3* rs1056892 polymorphism was discovered to be more prevalent in individuals who had cardiac damage following anthracycline therapy than in a control group by D. L. Hertz et al. in 2015 [34]. Overall, 166 women treated for breast cancer were included, while 19 of them were found to have worsened systolic heart function (EF decreased lower than 55 percent). Interestingly, the *CBR3* rs1056892 variant was found to be more common in patients who developed a heart injury than in a control group, showing that this *CBR3* gene polymorphism actually significantly increases the risk of cardiotoxicity (OR = 2.50, 95% CI 1.22–5.11, *p* = 0.012), as it was thought more than ten years ago by Blanco et al. mentioned above [35,36]. The *CBR3* gene rs1056892 polymorphism had a substantial correlation with ABCC in breast cancer patients according to the 2017 Serie et al. research [37]. The researchers strongly agreed that their study repeatedly showed that the *CBR3* gene rs1056892 has a significant association with CHF (*p* = 0.004) in breast cancer patients [37].

### 3.6. CBR1 rs9024

The results revealed that the AA genotype was associated with a decreased risk of ABCC; however, due to the small number of varied versions, we were unable to draw any conclusions. There is a scarcity of data on *CBR1* rs9024 in the literature. Lombrana and colleagues examined cardiac tissues from patients with and without Down syndrome who had been given anthracycline treatment. The study discovered a 1.6-fold increase in CBR1 expression in Down syndrome individuals as well as the fact that this protein is responsible for around 90% of daunorubicin metabolism. Higher quantities of a potentially toxic anthracycline metabolite may arise in cardiomyocytes, resulting in cardiac damage [38]. Other researchers were unable to demonstrate a substantial influence of the *CBR1* SNP on ABCC in children. Data from phase II research examining the pharmacokinetics of doxorubicin in 100 children were evaluated; however, *CBR1* rs9024 had no significant influence [39].

### 3.7. NCF4 rs1883112

NCF4 is a multicomponent enzyme system variation that causes an oxidative burst in which electrons are transferred from NADPH to molecular oxygen, resulting in reactive oxidant intermediates [19]. The involvement of the NAD(P)H oxidase in ABCC was verified in mice deficient in the gp91 subunit of this enzyme; the gp91 mutants exhibited a reduced NAD(P)H oxidase activity in heart homogenate [40,41]. Anthracyclines, a class of chemotherapeutic agents, are known to induce cardiotoxicity primarily through the overproduction of ROS, leading to oxidative stress and subsequent cardiac damage. Given NCF4’s role in ROS generation, variations in this gene could potentially influence an individual’s susceptibility to anthracycline-induced cardiotoxicity [42]. The kinetics of a chronic doxorubicin effect was first established in wild-type mice; repeated intraperitoneal injections of doxorubicin resulted in a severely impaired cardiac function [43]. In our example, we have acute ABCC population research, and we observe a statistically significant risk reduction in AA and AG carriers compared to GG carriers in univariate logistic regression analysis. In multivariate logistic regression analysis, the AG vs. GG variant shows a tendency for risk reduction in ABCC without statistical power. For acute ABCC, our data and Wojnowsi’s data demonstrate that this SNP has a mixed function in the development of ABCC. It is probable that fibrosis is not a significant pathological feature in the early stages; therefore, the NCF4 rs1883112 polymorphism has a varied influence in various ABCC stages. Direct NCF4 rs1883112 influence on fibrosis becomes crucial in the late phase of cardiotoxicity, when fibrosis is the most recognized hallmark of cardiotoxicity [44]. In a study of 97 consecutive decedents with a cancer diagnosis (48 of whom were treated with anthracyclines), cardiac histological lesions and *NCF4* rs1883112 were shown to be highly linked with cardiac fibrosis [45]. The findings of Wojnowski’s study on this SNP are highly fascinating. The effect varies depending on the stage of ABCC. In the case of chronic/late ABCC, *NCF4* rs1883112 plays a key role in increasing the risk for ABCC via the fibrosis formation mechanism, and it has a statistically significant risk increase for late ABCC. When they look at its impact on ABCC in the acute phase (as in our study), the results are not as compelling. The OR for acute ABCC following anthracycline therapy ranges from a little decrease to a slight rise [44]. For instance, the NCF4 GG genotype has been linked to a reduced risk of developing cardiotoxicity among Non-Hodgkin lymphoma patients treated with anthracyclines. This suggests that genetic variations in NCF4 may modulate the extent of ROS production during anthracycline therapy, thereby influencing cardiotoxic outcomes [44].

### 3.8. CYBA rs1049255

CYBA is an NADPH-oxidase, a multicomponent enzyme system variant. The SNP rs1049255 is found in the *p22phox* (*CYBA*) gene, which codes for the major subunit of the NADH/NADPH-oxidase [20]. We wanted to see if rs1049255 had any influence on ABCC risk. Our findings were negative, indicating that *CYBA* rs1049255 had no effect on ABCC risk. There is no research on rs1049255 for ABCC that has been published. However, research that analyzed the Iranian population [20] found that the rs10492255 and rs4673 variations are comparable in different cardiac pathologies. In the ABCC field, Wojnowski et al. examined *CYBA* rs4673 variation. There were no connections between this SNP’s role in ABCC [44].

### 3.9. Limitations of This Study

This study has several limitations that should be acknowledged. First, the relatively small sample size (*n* = 81) limited the statistical power to detect associations and constrained the ability to perform more advanced modeling techniques, such as penalized regression or external validation. As a result, our findings should be interpreted as exploratory and hypothesis-generating rather than definitive.

Second, although we included key cardiovascular risk factors in multivariate models, we were unable to fully adjust for other potentially important confounders, such as lifestyle factors (e.g., diet, physical activity), concomitant medications, and medication interactions. This limitation may have introduced residual confounding and biased some of the observed associations between genetic variants and subclinical cardiotoxicity. Future studies with larger and more diverse populations are needed to better account for these variables.

Third, heterogeneity in chemotherapy regimens and supportive treatments (e.g., use of trastuzumab, taxanes) may have influenced cardiotoxicity outcomes. Although we evaluated regimen distribution and found no statistically significant differences between groups, the influence of combination therapy cannot be completely excluded. Additionally, drug–gene interactions could not be comprehensively assessed, and pharmacokinetic data were not available.

Fourth, echocardiographic assessments of left ventricular ejection fraction (LVEF) were performed according to guidelines; however, inter-observer variability and limited sensitivity of conventional 2D echocardiography may affect the precision of subclinical damage detection. Future studies employing advanced imaging techniques, such as cardiac MRI or strain imaging, may provide greater diagnostic accuracy.

Lastly, this was a single-center study focused on breast cancer patients. Broader generalizability to other malignancies or treatment settings may be limited. Nevertheless, as one of the few prospective studies addressing subclinical anthracycline-induced cardiotoxicity in the context of pharmacogenomics, these results offer valuable insights and a foundation for further investigation.

## 4. Materials and Methods

### 4.1. Study Population

This prospective study was conducted from March 2015 to September 2020 at the Lithuanian University of Health Sciences (LUHSH) Kaunas Clinics, involving the Departments of Oncology and Cardiology. Ethical approval was granted by the Kaunas Regional Bioethics Committee (approval numbers: BEC MF 361, BE-2-10, and P1-BE-2-10/2014). The analysis included data from 81 breast cancer patients who received doxorubicin-based chemotherapy at the LUHSH Kaunas Clinics outpatient facility. The sample size was calculated using Kelsey’s formula. However, all analyses performed were underpowered to detect modest allele frequency differences, and any observed associations should be considered exploratory. All participants provided written informed consent prior to enrollment and before undergoing two-dimensional echocardiography (2DE). Eligibility was assessed based on defined inclusion and exclusion criteria. Patients qualified for the study if they met the following conditions: (1) diagnosed with stage I to III nonmetastatic breast cancer; (2) eligible for chemotherapy regimens including conventional doxorubicin; (3) normal systolic and diastolic cardiac function on echocardiography prior to treatment; (4) absence of heart failure symptoms and baseline left ventricular ejection fraction (LVEF) ≥ 55%; (5) well-managed blood pressure, requiring no more than two antihypertensive medications and classified as arterial hypertension grade ≤ 2. Exclusion criteria included (1) presence of significant cardiovascular conditions (such as heart failure, arrhythmias, valvular disease, myocardial infarction, stroke, or angina); (2) contraindications to chemotherapy, including severe renal impairment (GFR < 30 mL/min) or other notable medical conditions as judged by the treating oncologist; (3) poorly controlled hypertension, identified via medical records and patient interviews (ABP ≥ 140/90 mmHg); (4) presence of metastatic disease; and (5) withdrawal of consent at any point during the study. Additional medical background was obtained from general practitioner referrals and clinical documentation. The cohort was divided into two subgroups: individuals who developed subclinical cardiac injury (Group *n* = 30) and those without such findings (Control group *n* = 51). SNPs were selected after literature review and considering other preclinical and clinical studies, keeping a focus on drug metabolism pathways.

Chemotherapy regimen distribution is detailed in Table 4. Chemotherapy regimens were as follows:

- AC (Doxorubicin 60 mg/m^2^ IV plus cyclophosphamide 600 mg/m^2^ IV on day 1 every 3 weeks for four cycles);
- FAC (5-FU 500 mg/m^2^ IV on days 1 and 8 or days 1 and 4 plus doxorubicin 50 mg/m^2^ IV on day 1 plus cyclophosphamide 500 mg/m^2^ IV on day 1 every 3 weeks for six cycles);
- AC-paclitaxel (Doxorubicin 60 mg/m^2^ IV plus cyclophosphamide 600 mg/m^2^ IV on day 1 every 3 weeks for four cycles, followed by paclitaxel 80 mg/m^2^ by 1 h IV infusion weekly for 12 weeks or paclitaxel 175 mg/m^2^ every 21 days for four cycles);
- TAC (Docetaxel 75 mg/m^2^ IV plus doxorubicin 50 mg/m^2^ IV plus cyclophosphamide 500 mg/m^2^ IV on day 1 every 3 weeks for six cycles);
- FAC-docetaxel (5-FU 500 mg/m^2^ IV plus doxorubicin 50 mg/m^2^ IV plus cyclophosphamide 500 mg/m^2^ IV on day 1 every 3 weeks for three cycles, followed by docetaxel 100 mg/m^2^ IV every 3 weeks for three cycles);
- AC-docetaxel (Doxorubicin mg/m^2^ IV plus cyclophosphamide 600 mg/m^2^ IV on day 1 every 3 weeks for four cycles, followed by docetaxel 100 mg/m^2^ by 1 h IV every 21 days for four cycles).

In every protocol, doxorubicin was delivered via a 1-h intravenous infusion. Trastuzumab was combined with taxanes, followed by monotherapy, in accordance with standard adjuvant treatment guidelines for a total duration of 52 weeks. The administration of trastuzumab showed no statistically significant effect on the reduction in LVEF and was administered to 17 (21%) patients (*p* > 0.05).

### 4.2. Echocardiography Methods

Each participant underwent two-dimensional echocardiography (2DE) at four points: prior to chemotherapy initiation, during the 4th treatment week, at the conclusion of therapy, and six months after treatment completion. Imaging was performed using a Philips Epiq 7G (Philips Ultrasound, Inc. Koninklijke Philips N.V., Netherlands) ultrasound system, following established clinical guidelines. Left ventricular ejection fraction (LVEF) was assessed from apical two- and four-chamber views using the modified biplane Simpson method based on calculated left ventricular (LV) volumes. Subclinical cardiotoxicity due to anthracycline exposure was defined as a drop in LVEF of ≥10% from baseline to a value ≤ 55% in the absence of clinical symptoms or signs [46].

### 4.3. Genotyping Methods

Genetic analysis of SNPs related to drug metabolism was conducted at the Dr. K. Janušauskas Laboratory of Genetics, Institute of Biology Systems and Genetic Research, Lithuanian University of Health Sciences. TaqMan SNP Genotyping Assays (Applied Biosystems, Foster City, CA, USA) were utilized for SNP detection of the target genes. Genotyping was carried out using real-time polymerase chain reaction (PCR), PCR mixture composition is provided in Table 5. In 25 μL reaction mixtures consisting of 2 ng of template DNA, 12.5 μL of 2× TaqMan™ Universal Master Mix II without UNG, and 1.25 μL of 20× working stock derived from an initial 40× or 80× TaqMan SNP Genotyping Assay. Nuclease-free ddH_2_O was added to reach a total reaction volume of 25 μL. Each sample included 2 μL of DNA. Negative controls contained only nuclease-free ddH_2_O, and positive controls used DNA with known genotypes. To ensure reliability, all samples were genotyped in duplicate. Reactions were run on the Applied Biosystems 7900HT Real-Time PCR System. The thermal cycling protocol began with an initial denaturation at 95 °C for 10 min, followed by 40 amplification cycles (95 °C for 15 s and 60 °C for 1 min) as detailed in Table 6. Allelic discrimination was conducted using SDS 2.3 software from Applied Biosystems (Foster City, CA, USA).

### 4.4. Statistical Analysis

The distribution of all genotypes in cases and controls was assessed for consistency with Hardy–Weinberg equilibrium (HWE). The Student’s *t*-test was used to compare age across groups, and the Mann–Whitney U test was used to compare cumulative doxorubicin dosage. Pearson’s χ^2^ and Fisher’s exact tests were applied to assess allele and genotype frequency differences between cases and controls. Associations between SNPs and anthracycline-based chemotherapy cardiotoxicity (ABCC) were examined using univariate and multivariate logistic regression. To mitigate the risk of overfitting, a parsimonious modeling approach was applied by including only clinically relevant variables with prior biological plausibility and statistically significant univariate associations. The number of covariates in multivariate models was limited to maintaining a favorable events-per-variable ratio. Although penalized regression methods were not used due to the sample size, model robustness was supported through consistent trends across different inheritance models (allelic, genotypic, dominant, and recessive). Comparisons of homozygous versus wild-type + heterozygous and heterozygous + homozygous versus wild-type genotypes were made where applicable. Potential confounders, including lifestyle factors and concomitant medications, were assessed but not controlled in the final models due to sample size limitations. The results are presented as odds ratios (ORs) with 95% confidence intervals (CIs), and a two-tailed *p* value < 0.05 was considered statistically significant. All analyses were performed using SPSS v29 software.

## 5. Conclusions

Drug metabolism-related gene variants *SULT2B1* rs10426377, *UGT1A6* rs17863783, *CBR1* rs9024, *CBR3* rs1056892, *NCF4* rs1883112, and *CYBA* rs1049255 did not reach statistically important impact on ABCC in multivariate logistic regression analysis. However, we identified that *NCF4* rs1883112 had a risk reduction tendency for ABCC. Given the complex interplay between genetic variants and cardiovascular risk—where some SNPs may increase risk while others decrease it—large-scale, multicenter studies are needed to validate our findings and provide more conclusive evidence regarding the clinical implications of these genetic associations.

## Figures and Tables

**Table 1:** Cancer characteristics of the control individuals and patients.

Characteristics	All *n* = 81 (%)	Controls *n* = 51 (%)	ABCC Group *n* = 30 (%)	*p* Value
Age, years (mean ± SD)	54.11 ± 9.4	54.8 ± 8.9	52.9 ± 10.3	0.365
≤65 years	73 (90.1)	48 (94.1)	25 (83.3)	0.139
>65 years	8 (9.9)	3 (5.9)	5 (16.7)	
Arterial hypertension	32 (39.5)	9 (17.6)	23 (76.7)	<0.0001
Positive family history of cardiovascular disease	20 (24.7)	4 (7.8)	16 (53.3)	<0.0001
Diabetes mellitus	13 (16.0)	6 (11.8)	7 (23.3)	0.215
Dyslipidemia	19 (23.5)	9 (17.6)	10 (33.3)	0.108
Smoking status	16 (19.8)	8 (15.7)	8 (26.7)	0.231
Body mass index (BMI), kg/m^2^ (mean ± SD)	27.098 ± 5.599	27.288 ± 5.835	26.774 ± 5.255	0.692
<25 kg/m^2^	32 (39.5)	21 (41.2)	11 (36.7)	0.688
≥25 kg/m^2^	49 (60.5)	30 (58.5)	19 (63.3)	
**Pathological stage (pTNM)**				
0	3 (3.7)	2 (3.9)	1 (3.3)	0.483
IA	21 (25.9)	14 (27.5)	7 (23.3)	
IB	14 (17.3)	8 (15.7)	6 (20.0)	
IIA	27 (33.3)	18 (35.3)	9 (30.0)	
IIB	7 (8.6)	6 (11.8)	1 (3.3)	
IIIA	7 (8.6)	2 (3.9)	5 (16.7)	
IIIB	2 (2.5)	1 (2.0)	1 (3.3)	
**Breast side**				
left	33 (40.7)	19 (37.3)	14 (46.7)	0.678
right	47 (58.0)	31 (60.8)	16 (53.3)	
both	1 (1.2)	1 (2.0)	0 (0.0)	
**Differentiation**				
G1	1 (1.2)	1 (2.0)	0 (0.0)	0.531
G2	64 (79.0)	38 (74.5)	26 (86.7)	
G3	16 (19.8)	12 (23.5)	4 (13.3)	
Luminal A subtype	29 (35.8)	16 (31.4)	13 (43.3)	0.278
Luminal B subtype	28 (34.6)	18 (35.3)	10 (33.3)	0.858
HER2-enriched subtype	17 (21.0)	11 (21.6)	6 (20.0)	0.867
Triple negative subtype	13 (16.0)	10 (19.6)	3 (10.0)	0.353
**Estrogen receptor (ER)**				
negative (−)	26 (32.1)	18 (35.3)	8 (26.7)	0.422
positive (+)	55 (67.9)	33 (64.7)	22 (73.3)	
**Progesterone receptor (PR)**				
negative (−)	33 (40.7)	24 (47.1)	9 (30.0)	0.131
positive (+)	48 (59.3)	27 (52.9)	21 (70.0)	
**Human epidermal growth factor receptor 2 (HER2)**				
negative (−)	64 (79.0)	40 (78.4)	24 (80.0)	0.867
positive (+)	17 (21.0)	11 (21.6)	6 (20.0)	

**Table 2:** Distribution of drug metabolism-related gene variants and univariate logistic regression analysis between controls and ABCC group.

Allele/Genotype	Controls (*n* = 51)	ABCC Group (*n* = 30)	OR (95% CI)	*p* Value
***PON1* rs662**				
GG	3 (5.9)	1 (3.3)	0.517 (0.05–5.321)	0.579
AG	17 (33.3)	9 (30.0)	0.821 (0.307–2.196)	0.694
AA	31 (60.8)	20 (66.7)	1 (reference)	
GG, AG vs. AA			0.775 (0.301–1.993)	0.597
GG vs. AG, AA			0.552 (0.055–5.556)	0.614
***PON1* rs3735590**				
CT	6 (11.8)	0	NA	NA
CC	45 (88.2)	30 (100)	1 (reference)	
***SULT2B1* rs10426377**				
AA	7 (13.7)	3 (10.0)	0.857 (0.192–3.830)	0.840
AC	16 (31.4)	13 (43.3)	1.625 (0.614–4.301)	0.328
CC	28 (54.9)	14 (46.7)	1 (reference)	
AA, AC vs. CC			1.391 (0.563–3.439)	0.474
AA vs. AC, CC			0.698 (0.166–2.933)	0.624
***UGT1A6* rs17863783**				
TT	0 (0.0)	0 (0.0)	0 (0.0)	NA
GT	3 (5.9)	0 (0.0)	0 (0.0)	NA
GG	48 (94.1)	30 (100.0)	1 (reference)	
TT, GT vs. GG			NA	NA
TT vs. GT, GG			NA	NA
***CBR1* rs9024**				
AA	3 (5.9)	0 (0.0)	NA	NA
AG	11 (21.5)	9 (30.0)	1.442 (0.514–4.042)	0.487
GG	37 (72.5)	21 (70.0)	1 (reference)	
AA, AG vs. GG			1.133 (0.419–3.060)	0.806
AA vs. AG, GG			NA	NA
***CBR3* rs1056892**				
AA	9 (17.6)	5 (16.7)	0.556 (0.186–1.658)	0.292
AG	22 (43.1)	15 (50.0)	0.682 (0.354–1.314)	0.253
GG	20 (39.2)	10 (33.3)	1 (reference)	
AA, AG vs. GG			0.645 (0.368–1.132)	0.127
AA vs. AG, GG			0.933 (0.281–3.099)	0.910
***NCF4* rs1883112**				
AA	8 (15.7)	4 (13.3)	0.500 (0.151–1.660)	0.258
AG	27 (52.9)	13 (43.3)	0.481 (0.248–0.933)	**0.030**
GG	16 (31.4)	13 (43.3)	1 (reference)	
AA, AG vs. GG			0.486 (0.272–0.867)	**0.015**
AA vs. AG, GG			0.827 (0.226–3.020)	0.774
***CYBA* rs1049255**				
AA	16 (31.4)	11 (36.7)	0.688 (0.319–1.481)	0.339
AG	26 (51.0)	13 (43.3)	0.500 (0.257–0.973)	**0.041**
GG	9 (17.6)	6 (20.0)	1 (reference)	
AA, AG vs. GG			0.571 (0.346–0.944)	**0.029**
AA vs. AG, GG			1.266 (0.490–3.273)	0.626

**Table 3:** Multivariate logistic regression analysis of the SNPs between controls and ABCC group.

Covarities	OR	95% CI	*p* Value
***PON1 rs662***			
GG vs. AA	NA	NA	NA
AG vs. AA	0.724	0.246–4.946	0.804
Age > 65 years	7.268	0.498–89.614	0.186
Arterial hypertension	22.994	3.657–124.414	<0.0001
Cardiovascular disease in family history	25.046	4.821–159.841	0.001
Diabetes mellitus	3.258	0.421–24.614	0.154
Dyslipidemia	1.756	0.315–9.798	0.684
Smoking	0.684	0.048–4.978	0.594
≥25 kg/m^2^ Body mass index (BMI)	0.714	0.089–2.460	0.284
***PON1 rs3735590***			
CT vs. CC	NA	NA	NA
Age > 65 years	2.694	0.847–28.284	0.451
Arterial hypertension	19.983	3.546–79.159	<0.0001
Cardiovascular disease in family history	23.657	3.368–135.687	0.001
Diabetes mellitus	3.656	0.494–2.834	0.368
Dyslipidemia	1.219	0.213–8.343	0.484
Smoking	0.198	0.047–4.965	0.498
≥25 kg/m^2^ Body mass index (BMI)	0.651	0.023–2.552	0.367
***SULT2B1 rs10426377***			
AA vs. CC	0.084	0.005–1.350	0.080
AC vs. CC	0.788	0.169–3.678	0.762
Age > 65 years	6.638	0.599–73.604	0.123
Arterial hypertension	26.902	5.227–138.464	<0.0001
Cardiovascular disease in family history	25.046	3.780–165.944	0.001
Diabetes mellitus	3.043	0.524–17.662	0.215
Dyslipidemia	1.756	0.315–9.798	0.521
Smoking	0.502	0.063–3.978	0.514
≥25 kg/m^2^ Body mass index (BMI)	0.373	0.075–1.846	0.227
***UGT1A6 rs17863783***			
TT vs. GG	NA	NA	NA
GT vs. GG	NA	NA	NA
Age > 65 years	3.202	0.392–26.126	0.277
Arterial hypertension	16.196	3.806–68.922	<0.0001
Cardiovascular disease in family history	19.836	3.377–116.513	0.001
Diabetes mellitus	2.422	0.439–13.361	0.310
Dyslipidemia	2.364	0.464–12.042	0.300
Smoking	0.511	0.072–3.598	0.500
≥25 kg/m^2^ Body mass index (BMI)	0.507	0.113–2.267	0.374
***CBR1 rs9024***			
AA vs. GG	NA	NA	NA
AG vs. GG	1.492	0.301–7.398	0.624
Age > 65 years	3.087	0.365–26.116	0.301
Arterial hypertension	16.253	3.721–70.993	<0.0001
Cardiovascular disease in family history	19.357	3.239–115.671	0.001
Diabetes mellitus	3.027	0.473–19.355	0.242
Dyslipidemia	1.960	0.399–9.628	0.407
Smoking	0.448	0.064–3.144	0.419
≥25 kg/m^2^ Body mass index (BMI)	0.431	0.099–1.880	0.263
***CBR3 rs1056892***			
AA vs. GG	0.597	0.068–5.212	0.640
AG vs. GG	1.341	0.274–6.557	0.717
Age > 65 years	4.933	0.452–53.887	0.191
Arterial hypertension	19.214	4.442–83.107	<0.0001
Cardiovascular disease in family history	18.842	3.350–105.986	0.001
Diabetes mellitus	2.115	0.341–13.123	0.421
Dyslipidemia	1.774	0.339–9.272	0.497
Smoking	0.424	0.057–3.158	0.403
≥25 kg/m^2^ Body mass index (BMI)	0.431	0.095–1.957	0.276
***NCF4 rs1883112***			
AA vs. GG	0.297	0.027–3.304	0.323
AG vs. GG	0.312	0.067–1.461	0.139
Age > 65 years	3.399	0.400–28.863	0.262
Arterial hypertension	22.970	4.795–110.037	<0.0001
Cardiovascular disease in family history	21.796	3.493–136.028	0.001
Diabetes mellitus	3.164	0.506–19.785	0.218
Dyslipidemia	2.417	0.456–12.805	0.300
Smoking	0.548	0.075–4.016	0.554
≥25 kg/m^2^ Body mass index (BMI)	0.321	0.061–1.683	0.179
***CYBA rs1049255***			
AA vs. GG	3.829	0.443–33.095	0.222
AG vs. GG	0.933	0.129–6.729	0.945
Age > 65 years	2.454	0.313–19.210	0.393
Arterial hypertension	30.349	5.609–164.211	<0.0001
Cardiovascular disease in family history	26.937	3.524–205.899	0.002
Diabetes mellitus	3.019	0.474–19.231	0.242
Dyslipidemia	1.950	0.382–9.961	0.422
Smoking	0.346	0.044–2.713	0.313
≥25 kg/m^2^ Body mass index (BMI)	0.419	0.090–1.951	0.268

**Table 4:** Chemotherapy regimens.

Regimen	Patients, *n* = 81 (%)	Controls, *n* = 51 (%)	ABCC Group, *n* = 30 (%)	*p* Value
AC	8 (9.9)	5 (9.8)	3 (10.0)	1.000
AC-paclitaxel	53 (65.4)	31 (60.8)	22 (73.3)	0.251
AC-docetaxel	7 (8.6)	6 (11.8)	1 (3.3)	0.250
FAC-docetaxel	5 (6.2)	4 (7.8)	1 (3.3)	0.646
TAC	5 (6.2)	2 (3.9)	3 (10.0)	0.354
FAC	3 (3.7)	3 (5.9)	0 (0.0)	0.292
Doxorubicin cumulative dose (mg/m^2^), (median, min.–max.)	37.740 (129.000–303.200)	236.250 (129.000–303.200)	239.350 (146.340–291.000)	0.339

**Table 5:** PCR mixture composition.

Reagents	Volume for One Reaction (μL)
2× TaqManTM Universal Master Mix II, no UNG	12.5
20× TaqMan SNP Genotyping Assay stock	1.25
Nuclease free water	9.25
Total volume	23
DNA	2
Final volume	25

**Table 6:** Real-time PCR cycling conditions.

Real-Time PCR Stage	Times and Temperatures	Cycles
DNA polymerase activation	10 min for 95 °C	1
DNA melting	15 s for 95 °C	40
DNA annealing/extending	1 min for 60 °C	

## Data Availability

The data presented in this study are available on request from the corresponding author due to ethical reasons.
